# Supplementary material for: Genetic Manipulation of CB1 Cannabinoid Receptors Reveals a Role in Maintaining Proper Skeletal Muscle Morphology and Function in Mice
Source: Int J Mol Sci. 2022 Dec 9;23(24):15653. doi: 10.3390/ijms232415653 (PMC9779148; doi:10.3390/ijms232415653)
Supplement: Supplementary file 1 [file ijms-23-15653-s001.zip › ijms-2041570-supplementary updated.pdf]

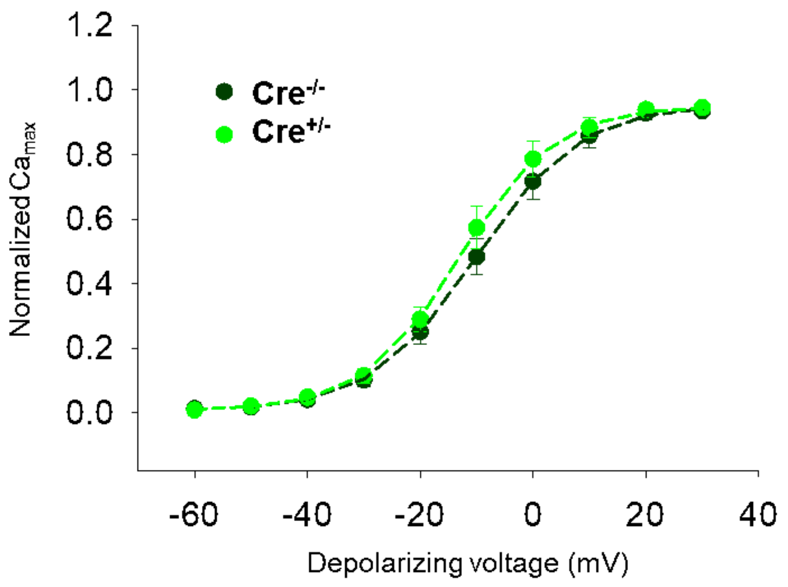

Figure S1. Ca_{max} values during repetitive 100 ms long membrane depolarizations applied from -60 to +30 mV on 21 and 27 FDB cells from $Cre^{+/-}$ and $Cre^{+/-}$ mice, respectively, normalized to the maximal change in calcium concentration measured on the given fiber. Ca_{max} was derived based on Eq. 1 detailed in Methods. Average values were fitted with a Boltzmann function (dashed curves) giving rise to the following parameters for $Cre^{-/-}$: V_{50}= -10.16 mV, k= 9.15 and Ca_{max} =0.39±0.05 μM for $Cre^{+/-}$: V_{50}=-13.13 mV, k=8.31 and Ca_{max}=0.40±0.04 μM.

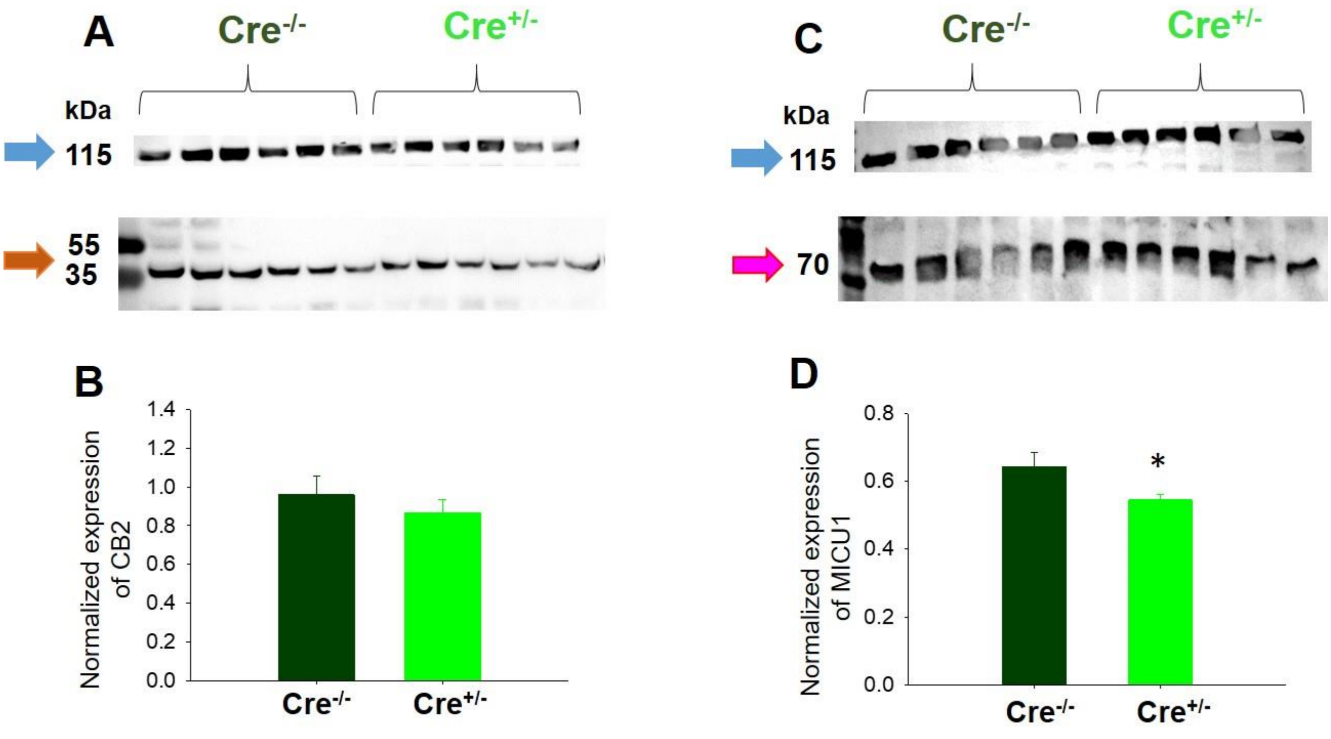

Figure S2. Western Blot analysis of TA muscle samples. Panels A and C are representative images illustrating the changes of protein expression for CB2 and MICU1 (orange and magenta arrows, 45 and 70 kDa) upon CB1 genetic manipulation and alfa-actinin (blue arrows, 115 kDa) that was used as internal control for normalizing purposes. Panels B and D are

the average normalized protein expression levels in the two samples for CB2 and MICU1, respectively. 6 animals per group were analyzed in at least 3 different independent experiments. *$p<0.05$

Nr. of pulses	F/F_0		Amount of Ca^{2+} released (μM)		Peak release flux (mM/s)		Steady flux (mM/s)	
	$Cre^{-/-}$	$Cre^{+/-}$	$Cre^{-/-}$	$Cre^{-/-}$	$Cre^{-/-}$	$Cre^{+/-}$	$Cre^{-/-}$	$Cre^{+/-}$
**Nr. of cells**	19	26	19	26	19	26	19	26
**1**	2.63±0.22	2.77±0.20	834.44±62.52	812.74±123.53	10.72±1.57	11.81±1.09	2.83±0.36	3.01±0.22
**2**	2.48±0.19	2.58±0.15	607.01±57.06	588.84±84.68	5.95±0.83	6.94±0.55	1.80±0.26	2.03±0.15
**3**	2.33±0.17	2.44±0.14	545.05±51.82	512.57±0.77	5.00±0.75	5.91±0.51	1.51±0.23	1.71±0.14
**4**	2.20±0.16	2.31±0.12	498.13±47.93	468.42±0.70	4.41±0.67	5.39±0.45	1.33±0.20	1.59±0.13
**5**	2.08±0.15	2.20±0.11	463.48±45.37	420.34±0.65	3.94±0.61	5.13±0.47	1.27±0.19	1.46±0.11
**6**	1.99±0.14	2.11±0.10	427.30±42.06	380.15±0.61	3.69±0.58	4.63±0.40	1.18±0.18	1.40±0.12
**7**	1.91±0.13	2.02±0.10	394.25±39.66	352.62±0.58	3.37±0.55	4.26±0.38	1.11±0.17	1.27±0.11
**8**	1.84±0.12	1.95±0.09	361.86±37.5	326.58±0.55	3.08±0.51	4.08±0.37	0.99±0.17	1.19±0.10

Table S1. Average parameters of the voltage induced calcium transients and calculated release fluxes during tetanic stimulation on FDB muscles.
